# Bikondylärer Oberflächenersatz des Kniegelenkes beim jungen Patienten – ein Update

**DOI:** 10.1007/s00132-021-04104-w

**Published:** 2021-04-09

**Authors:** Christian Egloff, Michael T. Hirschmann, Céline Moret, Philipp Henle, Martin Ellenrieder, Thomas Tischer

**Affiliations:** 1 Department of Orthopaedic Surgery and Traumatology, University Hospital Basel, Spitalstrasse 21/Petersgraben 4, 4031 Basel, Schweiz; 2 Department of Orthopaedic Surgery and Traumatology, Kantonsspital Baselland (Bruderholz, Liestal, Laufen), 4101 Bruderholz, Schweiz; 3 University of Basel, Basel, Schweiz; 4 Orthopädie Sonnenhof Bern, Bern, Schweiz; 5 Orthopädische Klinik und Poliklinik, Universitätsmedizin Rostock, Doberaner Straße 142, 18057 Rostock, Deutschland

**Keywords:** Untere Extremität, Mittleres Alter, Gonarthrose, Patientenzufriedenheit, Knietotalendoprothese, Lower extremity, Middle age, Osteoarthritis, Patient satisfaction, Total knee replacement

## Abstract

Die Zahl der durchgeführten Knietotalendoprothesen (KTEP) nimmt jedes Jahr kontinuierlich zu. Ungefähr 10 % davon betreffen Patienten unter 55 Jahren, obwohl bekannt ist, dass in dieser Altersgruppe die Zufriedenheit und die funktionellen Ergebnisse geringer und die Revisionsrate höher ausfällt. Vermehrte Aktivität und erhöhtes Anspruchsdenken machen die Endoprothetik in dieser Altersgruppe zu einer besonderen Herausforderung. Gleichzeitig ist der Anteil posttraumatischer Gonarthrosen deutlich erhöht, was in Anbetracht ligamentärer und knöcherner Vorschäden eine schwierigere operative Versorgung bedeutet. Bei fortgeschrittenen Arthrosen in mehreren Kompartimenten mit/ohne begleitender ligamentärer Instabilität muss jedoch ein totalendoprothetischer Ersatz auch bei jüngeren Patienten erwogen werden. Die sorgfältige Indikationsstellung für die KTEP und die eingehende Patientenaufklärung (Chancen, Risiken, erreichbare Leistungsfähigkeit in Beruf und Alltag) sind gerade bei jüngeren Patienten starke Prädiktoren für ein gutes Resultat.

## Abstract (en)

The absolute number of total knee arthroplasties (TKA) continues to rise every year. About 10% of the patients are less than 55 years of age, although it is known that functional results and patient satisfaction are lower combined with an increased likelihood of revision compared to older patients. Higher physical activity and patient expectations are a major challenge in this age group. At the same time, the incidence of posttraumatic/postoperative alterations is high, including ligamentous or bony deficiencies, which can make the surgical procedure challenging. In view of these facts conservative treatments and joint sparing procedures should always be considered first. The potential correction of lower-limb deformities and unicompartmental knee arthroplasties need to be carefully evaluated before considering total knee arthroplasty. Only in advanced cases of osteoarthritis in more than one compartment of the knee of with combined ligamentous instability, can a TKA provide satisfactory results in the young patient. However, the strongest predictor of satisfaction is a realistic expectation.

Durch die guten funktionellen Resultate, die potenziell langen Standzeiten und die hohe Zufriedenheit der Patienten sind gerade bei jüngeren Patienten die Erwartungen an die Technik der High-Performance-Gelenke sehr hoch. Damit verbunden ist die Hoffnung auf eine unbeeinträchtigte und quasi unveränderte Nutzbarkeit des ersetzten Gelenks – diese Hoffnung kann aber nicht immer erfüllt werden. Über diese bestehende Diskrepanz zwischen Erwartung und medizinisch erreichbarem Ergebnis muss sorgfältig aufgeklärt werden.

## Knietotalendoprothetik: Aktuelle Entwicklungen und Indikation

Durch die ausgezeichneten funktionellen Resultate, die Langlebigkeit der Implantate und die Zufriedenheit der Patienten sind die Erwartungshaltung und Hoffnungen gerade von jüngeren Patienten in die Technik der „High-Performance-Gelenke“ rasant gestiegen. Wir sehen daher auch weltweit einen immensen Anstieg dieser operativen Eingriffe. Projektionen aus den USA gehen sogar so weit, dass die Nachfrage für Knieprothesen von 2005 bis 2030 um 673 % Prozent steigen könnte [28]. Aktuell liegt in Deutschland und der Schweiz das Durchschnittsalter für eine Knietotalendoprothese (KTEP) bei knapp 69 Jahren, was auch ungefähr dem internationalen Vergleich entspricht [1, 6, 15, 39]. Die Ätiologie der Arthrose bei jungen Patienten unterscheidet sich jedoch entscheidend von der anderer Altersgruppen: Bei den jüngeren Patienten < 55 Jahren sind häufig Zustände nach schweren Traumata oder Deformitäten mit begleitenden ligamentären Instabilitäten oder Tumorresektion mit Substanzdefekt zu beobachten [13, 16].

**Figure 1:**
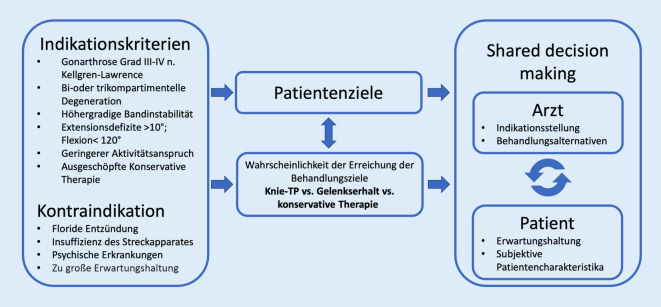

**Figure 2:**
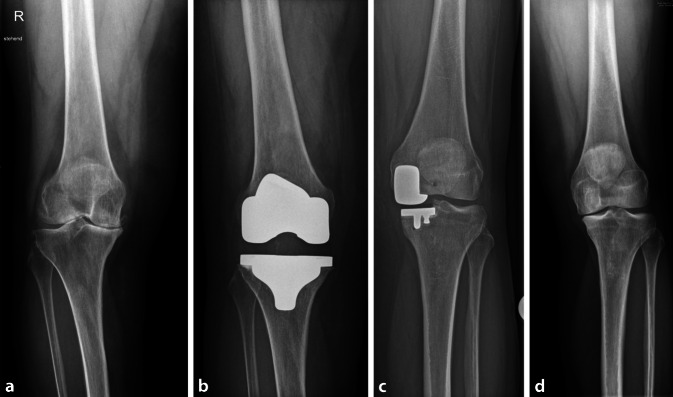

Die aktuelle Leitlinie der Arbeitsgemeinschaft der Wissenschaftlichen Medizinischen Fachgesellschaften (AWMF) berücksichtigt die Ziele des Patienten als einen zentralen Aspekt bei der Indikationsstellung zur KTEP (Abb. 1; [30]). Ein wichtiger Prädiktor für die Patientenunzufriedenheit nach einer KTEP sind nicht erfüllte Erwartungen (10-fach höheres Risiko) [8]. Die Patienten erwarten aufgrund ihres jungen Alters Schmerzfreiheit und eine quasi unveränderte Funktion und Stabilität eines gesunden Knies, sodass der Wiederaufnahme sportlicher und beruflicher Aktivitäten nichts mehr im Wege steht. Damit besteht eine relevante Diskrepanz zwischen Erwartung und dem aus ärztlicher Sicht erreichbaren Ergebnis (Abb. 2).

## Outcome nach bikondylärem Oberflächenersatz

Untersuchungen an Patienten < 55 Jahren weisen auf eine deutlich erhöhte Revisionsrate nach primärer KTEP im Vergleich zu älteren Patienten hin (15-Jahres-Revisionsrate von 15,5–18,2 % versus 2,6–4,6 % bei > 75-Jährigen) [1, 15, 39]. Auch innerhalb dieser Altersgruppe (< 55 Jahre) konnte ein Anstieg der Revisionsraten mit sinkendem Alter festgestellt werden. So mussten sich Patienten unter 40 Jahren 1,6-mal häufiger Revisionen unterziehen als 40- bis 54-Jährige [35]. Die Revisionsrate nimmt somit mit zunehmendem Alter kontinuierlich ab [22, 39].

Erlauben die knöchernen und ligamentären Strukturen die Implantation einer kreuzbanderhaltenden TEP, zeigen diese über alle Altersgruppen hinweg, bezüglich der Revisionsrate gegenüber der posterior stabilisierenden TEP in den Registern bessere Ergebnisse (8 % Revisionsrate bei kreuzbanderhaltender TEP vs. 11 % bei posterior stabilisierender TEP nach 15 Jahren) bei vergleichbaren funktionellen Resultaten [2, 39, 41]. Bei höheren Kopplungsgraden (teilgekoppelt oder Scharnierendoprothesen) erreichen die klinischen Ergebnisse mit einem Knee Society Score von durchschnittlich 85 Punkten und Standzeiten von 83 % nach 10 Jahren nicht die Ergebnisse des ungekoppelten Oberflächenersatzes (Knee Society Score von 92 Punkten, Standzeit 94 % nach 15 Jahren) [21, 31, 39, 40]. Diese Ergebnisse aus den Registern spiegeln sich über alle Altersgruppen hinweg. Bei jüngeren Patienten sollte man daher den minimal notwendigen Koppelungsgrad verwenden (Abb. 2).

Durch den Einsatz von ultrahochvernetztem Polyethylen („cross-linked polyethylene“ [XLPE]) konnte man den mechanisch induzierten Abrieb stark reduzieren [12, 32, 35]. Obwohl mobile Gleitlager („rotating polyethylen“) speziell zur Stressreduktion an der Tibia zur Reduktion des Polyethylenabriebs entwickelt wurden, zeigten sie bislang keine Vorteile in Bezug auf das Abriebverhalten, die Standzeiten oder die funktionellen Resultate [24, 25, 34]. Sie führten im Gegenteil in Schweden sogar zu 20 % erhöhten Revisionsraten – unabhängig vom Alter der Patienten [35].

In den skandinavischen und englischen Registern wird die höhere Revisionsrate bei jüngeren Patienten bei unzementierten im Vergleich zu zementierten Prothesen deutlicher als bei Älteren [1, 35, 39]. Mit ursächlich scheint die traditionell geringe Erfahrung mit zementfreien KTEP in diesen Ländern; zum Teil werden weniger als 10 % der KTEP zementfrei eingesetzt [35, 39]. Dagegen zeigte eine Metaanalyse von randomisierten Studien mit hoher Fallzahl keinen Unterschied bezüglich den funktionellen Resultaten, aseptischen Lockerungen oder Schmerzen bei Patienten unter 60 Jahren [11]. Somit kann zur Zementierungstechnik gerade bei jungen Patienten aktuell noch keine eindeutige Empfehlung abgegeben werden.

## Versagensgründe für die KTEP beim jungen Patienten – spezifische Aspekte

### Aseptische Revision

**Figure 3:**
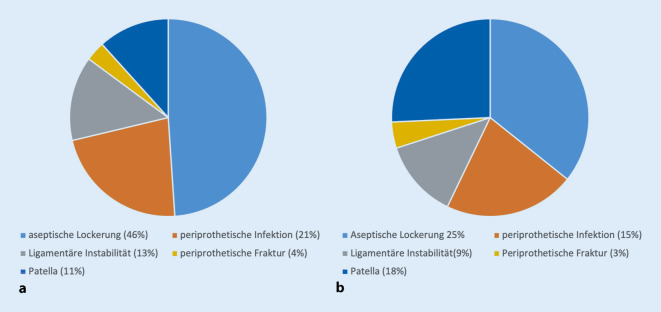

In der Subgruppe der unter 55-Jährigen konnte ein systematisches Review zeigen, dass fast die Hälfte der Revisionen aufgrund von aseptischen Lockerungen zustande kommt (46 %), gefolgt von den Infektionen (21 %) und der Instabilität (13 %) [3]. Das entspricht deutlich mehr Lockerungen als in anderen Altersgruppen publiziert wird ([1, 6, 15, 35, 39]; Abb. 3). Eines der wichtigsten spezifischen Risiken bei jungen Patienten nach Knietotalprothesenimplantation ist die Gestaltung ihrer körperlichen Aktivität. Während bei Camus und Kollegen [9] ein klarer Zusammenhang zwischen Aktivität und dem Auftreten einer aseptischen Lockerung bestand, zeigen andere Studien gegensätzliche Resultate: Huch et al. [18] fanden hinsichtlich der Langzeitergebnisse (Follow-Up ≥ 5 Jahre) keinen negativen Einfluss von „Non-Impact“-Sportarten auf das Versagensrisiko. Die Studie zeigte jedoch auch, dass nur 32 % der Patienten nach Knie-TEP-Implantation noch sportliche Aktivitäten ausübten, direkt vor der Operation gaben dies noch 42 % an.

Neben der erhöhten Aktivität der jüngeren Patienten scheint die Adipositas (BMI > 30 kg/m^2^) ein weiterer Risikofaktor gerade bei jüngeren Patienten zu sein. Einerseits zeigen KTEP adipöser Patienten generell signifikant kürzere Standzeiten und ein höheres Revisionsrisiko als die nichtadipöser Patienten (erhöhte Rate der aseptischen Lockerungen), andererseits zeigt sich auch eine erhöhte Prävalenz der morbiden Adipositas (BMI > 40 kg/m^2^) bei Patienten jünger als 50 Jahre mit einer Knieendoprothese [1, 10].

### Traumatische Komplikationen

Periprothetische Frakturen sind mit 4,7 % insgesamt generell ein eher seltener Revisionsgrund für KTEP [37]. Eine differenzierte Analyse des Mayo Clinic Total Joint Registry im Jahr 2013 konnte eine U‑förmige Altersverteilung für das Risiko einer periprothetischen Fraktur zeigen [38]. Das höchste Risiko tragen demnach die unter 60- sowie die über 80-Jährigen. In diesen beiden Gruppen ist das relative Risiko für eine periprothetische Fraktur etwa doppelt so hoch wie in den Gruppen von 60–70 und 70–80 Jahren. Eine Erklärung hierfür könnte in der höheren Aktivität und Risikobereitschaft der jüngeren Patienten liegen.

### Implantatassoziierte Infektion

Für diverse Risikofaktoren, z. B. Komorbiditäten wie Diabetes mellitus, ist der Zusammenhang zwischen Infektrisiko und Vorhandensein des Risikofaktors gut untersucht. Obwohl die implantatassoziierte Infektion die häufigste Ursache für einen frühzeitigen (< 2 Jahre) Knieprothesenwechsel darstellt, ist wenig über den Zusammenhang zwischen Infektionsrate und Patientenalter bekannt. Jedoch konnte eine populationsbasierte Studie (USA) aus dem Jahr 2014 eine deutliche Altersabhängigkeit für das Risiko einer implantatassoziierten Infektion innerhalb eines Jahres nach KTEP-Implantation darstellen [31]. Verglichen mit der Gruppe der über 64-Jährigen hatten die 50- bis 64-Jährigen ein 1,20fach erhöhtes Risiko für das Auftreten einer Frühinfektion, die unter 50-Jährigen gar ein 1,81fach erhöhtes Risiko. Die Autoren geben als eine mögliche Erklärung die höhere Prävalenz von posttraumatischen Arthrosen in der jungen Patientengruppe an. Vorangegangene Arthrotomien und die vorherige Verwendung von Fremdmaterial erhöhen bekanntermaßen das Infektionsrisiko bei nachfolgender Endoprothesenversorgung [31]. Zudem sind solche Eingriffe häufig komplex und mit längeren Operationszeiten sowie vermehrtem Blutverlust verbunden, was das Infektrisiko weiter ansteigen lässt. Bei jeglichem Verdacht auf einen („low-grade“) Infekt nach Voroperation oder einen Infektverlauf nach einer Voroperation in der Anamnese sollte die Entnahme mikrobiologischer und histologischer Proben mittels Probeexzision im Vorfeld einer KTEP-Implantation durchgeführt werden.

## Technische Besonderheiten beim jungen Patienten

In den letzten Jahren gab es einige zunächst vielversprechende Neuerungen: Computernavigation, Robotik, patientenspezifische Implantate, „high flex“-Knie, „gender knee“, antiallergische und abriebfeste Beschichtungen, diverse minimalinvasive Zugänge, neue Nachbehandlungskonzepte („fast-track“), neue Alignmentkonzepte (mechanisches versus kinematisches Alignment), neue Materialien (XLPE). Bezüglich der genannten Neuerungen bleibt es meist bei größeren Fallserien, jedoch fehlt die klare Evidenz zugunsten eines bestimmten Verfahrens.

Die computergestützte Navigation ist nach wie vor umstritten [23]. Vor allem weniger erfahrene Operateure können von der Navigation profitieren, um das hinsichtlich der Langzeitergebnisse günstige Maß (±3° um die neutrale femorotibiale Achse) zu treffen [5]. Ein optimales mechanisches Alignment führt jedoch nicht zu besseren klinischen (Langzeit‑)Resultaten. Aktuelle Reviews zeigen für das kinematische Alignment mindestens gleichwertige oder bessere funktionelle Ergebnisse und Komplikationsraten als für das mechanische Alignment [5, 36]. Es ist jedoch zu berücksichtigen, dass gerade bei jüngeren Männern in etwa einem Drittel ein Genu varum vorliegt [7]. Ein ausgeprägtes Varusalignment der tibialen Komponente könnte dann einen erhöhten, asymmetrischen PE-Abrieb begünstigen, als potenzielle Ursache für eine spätere Lockerung [20]. Jedoch fanden Howell et al. für die mittelfristigen Standzeiten (6,3 Jahre) nach kinematischem KTEP-Alignment außerhalb des ±3°-Korridors noch keine erhöhte Versagensraten [17]. Vor allem für jüngere KTEP-Patienten wird somit zukünftig nicht nur die simple anatomische Ausrichtung der Schnittlinien maßgebend sein. Die Berücksichtigung der individuellen Anatomie, Bandspannung, Kinematik und der Patientenansprüche bestimmt letztlich die Einstellung des Alignment [23]. So bleibt die operative Erfahrung ein wichtiger Faktor für die individuell optimale Einstellung des Alignment, v. a. beim jungen Kniearthrosepatienten mit (posttraumatischer) Deformität.

Gerade für junge Patienten sollten eher etablierte Verfahren verwendet werden

Gerade für junge Patienten sollten eher etablierte Verfahren verwendet werden, da sich frühe Fehlschläge neuer Technologien im jüngeren Alter besonders gravierend auswirken (Revisionsoperationen, Mehrfachwechsel). So ist z. B. bei posttraumatischen Fehlstellungen eine kombinierte knöcherne Achskorrektur (ein- oder zweizeitig) zu überlegen, um im späteren Verlauf Endoprothesen mit höherem Koppelungsgrad zu vermeiden. Auf teilweise schlechte Knochenqualität (z. B. posttraumatisch) oder die Folgen aseptischer Knochennekrosen im Jugend- oder Erwachsenenalter (Osteochondrosis dissecans, M. Ahlbäck) ist zu achten, vor allem bei Erwägung einer Versorgung mittels medialem Monoschlitten. Metallallergien werden ebenfalls mit verringerter Standzeit und schlechteren klinischen Ergebnissen nach KTEP bei jüngeren Patienten diskutiert. Bisher kann eine eindeutige Kausalität zwischen Frühlockerungen, chronischer Synovitis und Allgemeinsymptomen (u. a. Haarausfall) nach KTEP mit dem Vorliegen einer Metallallergie nicht hergestellt werden [29]. Vor dem Hintergrund der unsicheren Evidenzlage bleibt zusammenfassend die Empfehlung, im Falle einer Metallallergie die Implantatwahl, v. a. bei der Revision, anzupassen [19]. Zur Verfügung stehen grundsätzlich Implantatkomponenten ohne metallische Bestandteile (z. B. Aluminiumoxid oder Deltakeramik), mittels nichtallergener Materialien (z. B. Titan- oder ZrNb-Legierungen) beschichtete Komponenten oder oberflächenkeramisierte Implantate [4]. Bei der Implantatwahl ist zu berücksichtigen, dass diese Lösungsansätze auch Nachteile mit sich bringen. Keramikimplantate sind sehr teuer, zudem sind Brüche beschrieben [27]. Für oberflächenmodifizierte Implantate mit Zirkoniumoxid sind sowohl günstige Langzeitresultate für Patienten unter 50 Jahre beschrieben, aber auch Fälle des katastrophalen Versagens [14, 26]. Ein zukünftiger Ausweg könnten Modifikationen metallischer Femurkondylen sein, welche über mehrere Übergangsschichten letztlich in einer keramischen Oberfläche münden [33]. Klinische Langzeitresultate für derartig oberflächenmodifizierte Knieendoprothesen, vor allem für jüngere Patienten mit höherem Körpergewicht, Achsdeformitäten und gleichzeitig hohem Aktivitätsniveau, stehen jedoch aus.

## Fazit für die Praxis

- Wenn immer möglich, sollte beim jungen Patienten unter 50 Jahren der Gelenkersatz vermieden werden. Das biologische Potenzial des jüngeren Patienten erlaubt oftmals ein gelenkerhaltendes Verfahren mit guten Resultaten.
- Bei fortgeschrittenen primären und sekundären Arthrosen (z. B. postinfektiös, nach Trauma oder rheumatoider Arthritis) ist der endoprothetische Kniegelenkersatz zum Erhalt der Lebensqualität auch beim jungen Patienten indiziert und zeigt gute funktionelle Resultate.
- Die Erwartungshaltung des Patienten sollte präoperativ besprochen und mit realistischen Zielen verknüpft werden. Überzogene Erwartungen gelten als relative Kontraindikation.
- Aufgrund der durchschnittlich höheren körperlichen Aktivität beim jüngeren Patienten zeigen sich vermehrte Prothesenlockerungen. Dies kann in kürzeren Standzeiten, höheren Raten aseptischer Lockerungen und vermehrten Revisionen resultieren.
- Beim jungen Patienten sind die differenzierte Analyse der Grunderkrankung, des Alignements, der ligamentäre Stabilität und ein minimal notwendiger Kopplungsgrad essenziell für ein optimales Resultat.

## Abkürzungen

AWMF: Arbeitsgemeinschaft der Wissenschaftlichen Medizinischen Fachgesellschaften
BMI: Body-Mass-Index
KTEP: Knietotalendoprothese
PE: Polyethylen
TEP: Totalendoprothese
XLPE: „Cross-linked polyethylene“
